# Design of a mobile application for home monitoring of babies with complex congenital heart disease

**DOI:** 10.15649/cuidarte.2335

**Published:** 2023-03-29

**Authors:** David Castro, Paula Pérez-Rivero, Doris Cristina Quintero-Lesmes, Javier Castro

**Affiliations:** 1 . Fundación Cardiovascular de Colombia, Floridablanca, Colombia. Email: david.castroruiz@gmail.com Fundación Cardiovascular de Colombia Floridablanca Colombia david.castroruiz@gmail.com; 2 . Psychology Department, Health and Clinical Psychology Research Group. Universidad Pontificia Bolivariana, Bucaramanga Santander, Colombia Email: paula.perez@upb.edu.co Universidad Pontificia Bolivariana Universidad Pontificia Bolivariana Colombia paula.perez@upb.edu.co; 3 . Fundación Cardiovascular de Colombia, Floridablanca, Colombia. Email: dorisquintero@fcv.org Fundación Cardiovascular de Colombia Colombia dorisquintero@fcv.org; 4 . Fundación Cardiovascular de Colombia, Floridablanca, Colombia. Email: javiercastro@fcv.org Fundación Cardiovascular de Colombia Colombia : javiercastro@fcv.org

**Keywords:** Health, Technology, Pediatrics, Complex Congenital Heart Diseases, Home-Based Monitoring., Salud, Tecnología, Pediatría, Cardiopatías Congénitas Complejas, Monitoreo en Casa., Saúde, Tecnologia, Pediatria, Cardiopatias Congenitas Complexas, Monitoramento Domiciliar.

## Abstract

**Introduction:**

Congenital heart diseases are related to heart defects that develop during pregnancy and are present at birth. Children must regularly attend specialized clinical centers for treatment, which includes medical check-ups, hemodynamic procedures, and surgery. Nevertheless, the success of interventions largely depends on the education of parents and caregivers about the symptoms and warning signs that babies with complex congenital heart disease may present during the period between stages. Likewise, it is necessary for caregivers to have tools to face the barriers of the health system, such as, obstacles or delays in obtaining healthcare services. This facilitates timely health care for patients and avoids fatal outcomes or deterioration in quality of life.

**Materials and Methods:**

This document presents a system that provides support to patients with complex congenital heart disease in follow-up by a home monitoring program. The system consists of a mobile application that allows parents/caregivers to monitor physiological variables of the patients (weight, intake, and oximetry). The information registered in the application is sent for real-time evaluation by the medical team.

**Results:**

The application provides an effective means for sending data and communicating with the medical control center, in addition to offering educational and informative material. In this way, parents/caregivers get constant help and feedback about their baby's condition.

**Conclusions:**

The mobile application could help mitigate the costs of medical care and overcome the limitations of follow-up at home, giving doctors the possibility to see the patient's evolution and give timely recommendations.

## Resumen

**Introducción:**

las cardiopatías congénitas están relacionadas con defectos cardíacos que se desarrollan durante el embarazo y están presentes al nacer. El éxito de las intervenciones depende en gran medida de la educación de los padres/cuidadores sobre los signos y síntomas de alerta. Los cuidadores necesitan tener herramientas para hacer frente a las barreras del sistema de salud.

**Objetivo:**

crear un sistema como herramienta de seguimiento para los bebés con cardiopatías congénitas complejas que ayude a los padres/cuidadores y a los profesionales médicos a observar las variables fisiológicas del paciente y a brindar una retroalimentación oportuna y profesional a los padres/cuidadores desde la atención domiciliaria.

**Materiales y métodos:**

se presenta un sistema que proporciona apoyo a los pacientes que necesitan seguimiento de cardiopatías congénitas complejas mediante un programa de monitoreo en casa. El sistema consiste en una aplicación móvil que permite a los padres/cuidadores controlar las variables fisiológicas del paciente (peso, ingesta de alimento y oximetría). La información registrada en la aplicación se envía para ser evaluada en tiempo real por el equipo médico.

**Resultados:**

la aplicación proporciona un medio eficaz para enviar datos y comunicarse con el centro de control médico, además de ofrecer material educativo e informativo. De este modo, los padres/cuidadores reciben constantemente ayuda e información acerca del estado de su bebé.

**Conclusiones:**

la aplicación móvil podría ayudar a minimizar los costos de la atención médica y superar las limitaciones del seguimiento domiciliario, dando a los médicos la posibilidad de ver la evolución del paciente y dar recomendaciones oportunas.

## Resumo

**Introdujo:**

as doengas cardíacas congenitas estáo relacionadas a defeitos cardíacos que se desenvolvem durante a gravidez e estao presentes no nascimento. O sucesso das intervengóes depende em grande parte da educagáo dos pais/responsáveis sobre os sinais e sintomas de alerta. Os cuidadores precisam ter ferramentas para lidar com as barreiras do sistema de saúde.

**Objetivo:**

Criar uma ferramenta de sistema de monitoramento para bebes com doengas cardíacas congenitas complexas, que ajude pais/cuidadores e profissionais médicos a observar as variáveis fisiológicas do paciente e manter um feedback profissional e oportuno para os pais/cuidadores dos cuidados domiciliares.

**Materiais e Métodos:**

é apresentado um sistema que fornece suporte aos pacientes com cardiopatias congenitas complexas em acompanhamento através de um programa de monitoramento domiciliar. O sistema consiste em uma aplicagáo móvel que permite aos pais/responsáveis pelo acompanhamento das variáveis fisiológicas do paciente (peso, ingestáo e oximetria). As informales registradas no aplicativo sáo enviadas para serem avaliadas em tempo real pela equipe médica.

**Resultados:**

a aplicagáo fornece um meio eficiente para enviar dados e se comunicar com o centro de controle médico, além de oferecer material educativo e informativo. Desta forma, os pais/ responsáveis recebem ajuda constante e feedback sobre a condigáo de seu bebe.

**Conclusóes:**

a aplicagáo móvel pode ajudaraminimizaros custos do atendimentomédico e superaraslimitagóes do acompanhamento domiciliar, dando aos médicos a possibilidade de ver a evolugáo do paciente e darrecomendagóes oportunas.

## Introduction

Congenital heart defects are alterations in the structure and function of the heart present at birth^1^; these malformations are frequent in humans and are the second leading cause of death in children under one year of age in Colombia and were responsible for 22% of deaths between 2005 and 2013, according to the report of the Ministry of Health and Social Protection in 2015^2^.

Depending on their classification and medical criteria, some complex congenital heart defects may require two or more surgeries; the first being a stabilizing or palliation surgery. This first surgery often requires a high degree of investment in human and technological resources to be performed successfully, as in the case of Norwood surgery for children with Hypoplastic Left Heart Syndrome^3^. In the period between the first and second surgery, -first interstage period-, the baby remains at home under the care of her parents / caregivers and requires permanent and strict follow-up by her treating doctors. The goals for patients during this time at home are weight gain, prevention of infections and providing an environment that increases the emotional bond with their family. This period at home presents a challenge for parents / caregivers and the medical team, due to the high risk of complications and death, around 22% in some series.

This risk is currently recognized in multiple centers around the world and for this reason, health institutions have created monitoring^4^^-^^5^ and home supervision programs for these patients, which aim to create a bridge of constant communication between the medical team and parents/caregivers, which allows the periodic monitoring of the baby to detect signs and symptoms of risk, maintaining effective and permanent communication with health personnel, seeking that children survive and arrive in the best possible conditions to the second surgery ^4^^,^^6^^,^^7^.

This paper introduces the creation of a monitoring system tool for babies with complex congenital heart defects, which helps parents/caregivers and medical professionals to observe the physiological variables of the patient and maintain timely and professional feedback for parents/caregivers from home care.

## Materials and Methods

### System's General Architecture

The home monitoring system for children with complex congenital heart defects consists of a mobile application that allows the parent/caregiver to send data of their baby's physiological variables to a backend web using an API (application programming interface) that it is in charge of receiving, analyzing and saving the data in real-time.

**Figure 1:**
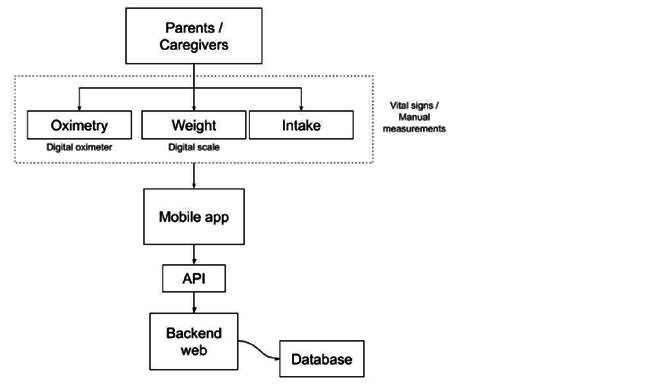
General System Architecture. Own elaboration

The parent/caregiver enters the data manually, after taking measurements with some external devices provided by the PROMESA project (Spanish acronym for: health education and monitoring program for children with complex congenital heart defects) to each patient. A digital oximeter (Masimo iSpO2 9577, Irvine, United States), a digital scale (Seca 334, Hamburg, Germany), and a smartphone with an active communications plan (Samsung Galaxy J5 Metal, Seoul, South Korea). Variables are sent periodically according to a previously established medical protocol, which is taught to parents/caregivers before their child leaves the hospital through a personalized education program that covers knowledge of the child's disease, medical-surgical procedures performed, monitoring plan, use of devices, care recommendations for the child and the caregiver. The oximetry data (oxygen saturation and heart rate), weight, and intake (breast or bottle) are sent from the mobile device to the backend web that receives, analyzes, and saves it for later viewing and analysis by the medical team. See Figure 1. 

Using the principles and practices of the Scrum framework^8^, a system was created that creates medical records for each patient in a database, allowing information to be stored and retrieved after logging into the phone application. The parents/caregivers manually enter the variables: weight, food intake, oxygen saturation, and heart rate, after measuring them according to the established protocol.

The data sent by the parents/caregivers are analyzed by the system (See Table 1) to determine if the patient is evolving adequately or if, on the contrary, it is on medical alert at the time of the analysis. This helps the medical team take the necessary medical decision for the patient that is being monitored.

**Table 1:** Evaluation of physiological variables. Medical alerts

Sign	Medical alert
Oxygen saturation	Oxygen saturation <75% Oxygen saturation > 90%
Heart rate	Heart rate < 90 per minute Heart rate >190 per minute
Daily weight	Failure to gain a daily weight < 20 grams for three consecu tive days (0.02 Kilograms) Daily weight reduction >30 grams (0.03 Kilograms) Sudden weight gains > 100 grams (0.1 Kilograms)
Feed volume	Breast milk: Number of intakes on the day <5 Bottle: reported feeding volume <100 ml/kg of Weight /day

If the parent/caregiver reports variables outside the limits established in each sign^9^, the backend web (control center) generates a graphic signal that indicates to the medical group that there is an alert with a patient, and they have the opportunity to take timely actions and/or give directions to the parent/ caregiver.*Qualitative research*

To evaluate usability, we carried out one group discussion with 10 caregivers of patients with complex congenital heart diseases. Caregivers had been used the application for six months (n=6), three months (n=2) and one month (n=2) depending on the patient's diagnoses timeline. Three investigators conducted data analyses. After the evaluation session, transcripts were generated, and investigators defined the topics that emerged during the discussion group with reference to field note data. Discrepancies regarding topics were to be resolved with a third party, but no discrepancies occurred.

Qualitative content analysis approach was used to code data, following the procedures of Hsieh & Shannon^10^. Specifically, we used conventional analysis, which facilitates gaining direct information from study participants without imposing preconceived categories^10^. First, investigators read each transcript from beginning to end. Then, open coding was carried out. Investigators read transcripts again, highlighting text that appeared to describe any experience or opinion regarding the app and writing in the margin of the text a keyword that capture the possible meaning. After open coding five transcripts, investigators decided on preliminary codes. Then they coded the remaining transcripts using these codes and adding new ones when encountered data that did not fit into an existing code. Saturation was established^11^ when all data from the session were classified under existing categories. A data set is created with the article information that can be consulted^12^. And you can download the app from play store^13^.

## Ethical considerations

This study counted with the approval of the Ethics Committee in Investigation of the Cardiovascular Foundation of Colombia and strictly followed the principles and ethical aspects contained in the last revision of the Declaration of Helsinki and the Resolution 08430 of 1993 of the Ministerio de Salud de Colombia.

## Results

**Figure 2:**
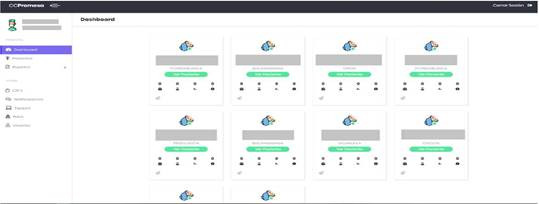
Backend web dashboard interface. Own elaboration

Figure 2 shows the main screen of the backend web, which is a dashboard that shows active patients, that is, those who are being followed at home, additionally each card shows if the baby has any alert for some evaluated sign.

Among the main functionalities of the backend are:

- Patient management.
- Viewing or downloading informative reports or graphics.
- The management of CRF's (case report forms) that can be applied to patients.
- The management of push notifications, which are sent to patients who are active in the follow-up.
- The management of the inventory of equipment delivered to parents/caregivers.

**Figure 3:**
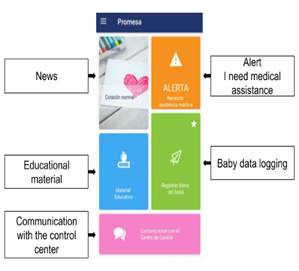
The main interface of the mobile application (Android) in Spanish.

Figure 3 shows the main screen of the mobile application (Android) Available to download from Google Play^13^. The main interface shows clear options that help parents/caregivers to understand the basic operation of the application. Since a high level of user-friendliness motivates users to maintain a commitment to the monitoring application^11^.

The main screen provides the parent/caregiver with the following options:

- Reading news of interest or educational material that includes, among other things, video and text components, which teach parents/caregivers about their child's illness and how to properly care for them, among other topics. (These contents have been developed specifically for the project with the direction and collaboration of the specialized medical team of the PROMESA project that includes pediatric cardiologists, pediatric hemodynamics, congenital cardiovascular surgeons, epidemiologists, psychologists, professional nursing.)
- Communication with the control center, which provides direct access to two direct channels with the medical team: a chat or a phone call.
- The alert button allows the parent/caregiver to send the medical team a notice indicating that their baby had to be taken to the nearest medical center in the emergency department, due to any eventuality or abnormality in their state of health.
- The patient data logging option, allows the parent/caregiver to enter and send the oximetry, weight, and intake data separately to the medical control center.

Additionally, the mobile application has internally a reminder functionality, which alerts the parent/ caregiver of the time when they should take a vital sign for follow-up. This is supported by push notification technology. TESTS

The testing phase was carried out to ensure the quality, efficiency, and effectiveness of the developed application. The monitoring system underwent two types of tests: unit and system. All the tests were carried out in conjunction with the medical team of the Cardiovascular Foundation of Colombia, who were given different profiles or types of users to carry out the tests, in some cases acting as parents/ caregivers with certain specific behaviors in the shipment of the information through mobile devices and others reviewing the backend web or control center to analyze the data sent by the parents/ caregivers and give the respective indications in each case.

Unit tests reviewed each module in isolation to ensure it met project requirements. To ensure the correctness of these applications, each functionality has been tested separately.

The system tests evaluated the integration of the different components of the application and their respective functionalities. To comprehensively test the system, a set of test cases were created. Table2 shows some of the most representative scenarios, which were used to test the integration of the entire system.

**Table 2:** Test cases

Test case	Scenarios
Send patient data (weight)	Create patient >> create mobile user >> mobile authentication >> register baby data >> weight
Send patient data (intake volume)	Create patient >> create mobile user >> mobile authentication >> record baby data >> intake volume
Send patient data (oximetry)	Create patient >> create mobile user >> mobile authentication >> register baby data >> oximetry
Send alert	Create patient >> create mobile user >> mobile authentication >> alert

### Qualitative results

All caregivers were mothers of patients with complex congenital heart diseases, aged 21 to 37 years old. They have used PROMESA application to register and send data about babies' progress (n=10), contact the nurse (n=6) get information about the disease and treatment (n=4) and informing an emergency (n=2). Participants who did not use application to contact the nurse referred they called her directly through the telephone line and the ones who did not look information commented they preferred to read about the disease in the parent's textbook. Regarding emergency events, caregivers said they called directly to the nurse when they were already in the hospital. Data were clustered in the following categories: ease of use, design, content, and utility.

### Ease of use:

All participants said they were able to use the application for sending data and that minimal assistance was required from the study team. One caregiver said: the true is that was very easy, I was worried about not being able to use the application, but then, I felt it was very friendly, for the data registration, you just follow some simple steps and that's it” (A, 37 years)

Minimal assistance was required one day because a general connection failure. Caregivers were informed by the study team and advised to record the data in their textbooks. S (28 years) said: “We all had a difficulty, I think there was a problem with the server for two days, actually it was not even a problem, the server was being checked, so we had to write the babies' information in our books, then the nurse called to tell me that I could send the data thorough the application"

### Design

All caregivers liked the app design regarding, colors, letters fond and style, distribution, placed of the bottoms, and alarms features. A mother stated: “the application is nice, the bottoms are very easy to find and use, visually is appealing, I like the colors and the images on it” (L, 24 years). Besides, the coherence of the design with other tools were stated several times: “you know the colors helped me to remember the steps for taking the measures, because the application used the same colors from the textbook, also used the same icons” (M, 32 years).

### Content

Caregivers provided positive feedback regarding content in the application. They liked having articles about disease, treatment, development milestones, mental health and practical recommendations for parenting in the context of congenital heart diseases: “every week we can find different information, for example, particularly I liked the articles about recipes and about stress management” (N, 27 years). However, only four caregivers referred using the app to get information, the other six, read in the textbook or on the website and social media of the project; “well at first I didn't know I can look the articles there, then the nurse told me, but I just get used to read in Facebook, so I never read the information in the app” (C, 29 years).

### Utility

The app was considered a useful tool to the caregivers especially because its design enhanced the register and sending process of important data about patients' progress. For instance, related to a question on the utility of the app a mother said:

“Yes, because it was very important to send the data on time, I guess, here, we all know that misinformation can cause our babies' death, that's why we needed an application that actually worked, like the one you have done.. .when the nurse received an abnormal record, immediately she called me, and that was possible because the app, was really very useful”

Caregivers stated that application was useful to record the daily patients' progress. The records were used to make decisions about treatment, but also for modifying diet, exercise (activity) and monitoring parameters: “the doctor always read the records, according to these, we follow the same or we adjust medications and diet” (S, 28 years).

## Discussion

The presented system, aids in the monitoring of patients with complex congenital heart defects by a specialized congenital heart diseases medical team, giving them the possibility to monitor the patient's physiological variables and respond effectively in the event of an eventuality or emergency, allowing them to issue expedient recommendations to parents/caregivers.

Among the possible limitations in our country for the mobile application presented in this document, it is that not all the Colombian geography has access to a 3G or 4G type cellular signal and the mobile app works through the use of the mobile telephone network, leaving out a significant number of the potential patients who are residents of rural areas of the country with limited or null access to the network.

Additionally, some areas of the country suffer from armed conflict, or in some cases, there have been cultural limitations since some parents/caregivers belong to ethnic groups, ideologies, or religions that prevent them from using certain technologies. On the other hand, in the former conflict scenario, the PROMESA program could be an opportunity to enable follow-up.

Therefore, the mobile application can be improved from several points of view, the first would be using GSM-type cellular networks to open use to patients living in limited areas. The second would be taking advantage of the Internet of Things (IoT) concept, which allows giving more reliability and precision to the data measured through the use of instruments connected directly to the mobile device, avoiding manual measurements throughout the methodology for taking physiological variables, enriching and improving the current version of the application.

Although entry into the project mentioned in the document (PROMESA) requires a level of literacy for the use of the technologies presented, experience has shown that it can be overcome thanks to the motivation of the parents/caregivers of the patients and the education program that accompanies the project, which parents/caregivers must complete previous to start monitoring from home, usually in hospital during the post operatory period of their child.

Caregivers' gave a generally positive feedback of the app. Regarding ease of use, content, design and utility the app was described as a tool that enhanced data recording and communication with the medical team. Azhar^14^ found similar results that indicated that even communication process and caregivers' skills were improved by using app tools and other technological devices.

Participants stated the information management as one of the main advantages of the app. Possibly, communication improves because the efficiency of the process is granted through the app. Besides, the fact that caregivers can access to information not only through articles content, but to the information given by the healthcare team is particularly important for the role they assume. According to Sherbourne and Stewart^15^ information and external support net are determinant for the prevention of mental fatigue in caregivers. Hence, monitoring programs for caregivers should include strategies for enhance communication and access to medical support^16^.

Related to the misuse of communications bottoms and articles included in the app, Gramszlo et al.^17^ found that caregivers are reluctant to involve in app use, because they consider this activity a distraction and a deviation from the time and attention their children require. These findings point out the importance of customizable app that compliance the lifestyle of caregivers and their families.

## Conclussion

The mobile application, together with a properly structured education and support program, could help mitigate the costs of medical care and overcome the limitations of home follow-up, giving the specialized medical team the possibility to watch patient's evolution and make timely recommendations. Furthermore, these types of applications and monitoring structures represent a change in medical care and can help reduce current limitations by providing a low-cost, continuous monitoring and communication with these patients with complex congenital heart defects, facilitating the opportune consultation that allows to quickly attend to a complication in progress, that can lead to the deterioration and death of these highly fragile children. Finally, these monitoring tools/educational programs could be adapted to other multiple pathologies and clinical scenarios with potential benefits for these patients.
